# Phase-Selective Synthesis of Anatase and Rutile TiO_2_ Nanocrystals and Their Impacts on Grapevine Leaves: Accumulation of Mineral Nutrients and Triggering the Plant Defense

**DOI:** 10.3390/nano12030483

**Published:** 2022-01-29

**Authors:** László Kőrösi, Balázs Bognár, Gyula Czégény, Simone Lauciello

**Affiliations:** 1 Research Institute for Viticulture and Oenology, University of Pécs, Pázmány P. u. 4, H-7634 Pécs, Hungary; 2 Institute of Organic and Medicinal Chemistry, Faculty of Pharmacy, University of Pécs, Szigeti st. 12, H-7624 Pécs, Hungary; balazs.bognar@aok.pte.hu; 3 Department of Plant Biology, University of Pécs, Ifjúság u. 6, H-7624 Pécs, Hungary; czegeny@gamma.ttk.pte.hu; 4 Electron Microscopy Facility, Istituto Italiano di Tecnologia, Via Morego 30, 16163 Genova, Italy; Simone.Lauciello@iit.it

**Keywords:** anatase, rutile, sol-gel, photocatalysis, reactive oxygen species, *Vitis vinifera*, grapevine, Cabernet Sauvignon, vitamin B_6_, pyridoxine, chlorophyll fluorescence

## Abstract

Titanium dioxide nanocrystals (TiO_2_ NCs), through their photocatalytic activity, are able to generate charge carriers and induce the formation of various reactive oxygen species (ROS) in the presence of O_2_ and H_2_O. This special feature makes TiO_2_ an important and promising material in several industrial applications. Under appropriate antioxidant balancing, the presence of ROS is crucial in plant growth and development, therefore, the regulated ROS production through the photocatalytic activity of TiO_2_ NCs may be also exploited in the agricultural sector. However, the effects of TiO_2_ NCs on plants are not fully understood and/or phase-pure TiO_2_ NCs are rarely used in plant experiments. In this work, we present a phase-selective synthesis of TiO_2_ NCs with anatase and rutile crystal phases. The nanomaterials obtained were characterized by means of X-ray diffraction (XRD), transmission electron microscopy (TEM), diffuse reflectance UV-Vis spectroscopy, and electron paramagnetic resonance spectroscopy (EPR). In field experiments, *Vitis vinifera* cv. Cabernet Sauvignon leaves developed under natural sunlight were treated with aqueous dispersions of TiO_2_ NCs at concentrations of 0.001, 0.01, 0.1, and 1 *w*/*v*%. The effect of the applied nanocrystals was characterized via leaf photochemistry, mineral nutrient contents, and pyridoxine levels. We found that stress responses of grapevine to anatase and rutile NCs treatments are different, which can be related to the different ROS profiles of the two polymorphs. Our results indicate that TiO_2_ NCs may be utilized not only for direct pathogen inactivation but also for eliciting plant defense mechanisms.

## 1. Introduction

Nowadays, research on engineered nanomaterials with unique physical and chemical properties is rapidly growing, which is clearly indicated by a large number of publications and patents [1,2]. The related scientific work is focused on two main fields. On the one hand, great efforts have been devoted to exploring nanomaterials with novel properties and exploiting their advantages in various fields [3,4]. On the other hand, the impact of nanomaterials on living organisms including plants and humans has also been investigated intensively [5,6,7]. Although the application of nanomaterials can revolutionize the old technologies, the threshold between their beneficial effects and their potential toxicity has yet to be explored.

Titanium dioxide (TiO_2_) is one of the most commonly used semiconductor metal oxides. Millions of tons of TiO_2_ are produced year by year. It is widely utilized as a white pigment in paints, foods, cosmetics, and pharmaceuticals [8]. Photocatalytic activity of TiO_2_ and the related photoinduced processes have attracted the attention of many researchers [9]. Its unique high-photoreactivity can be exploited in the field of heterogeneous photocatalysis for the removal of pollutants, in the production of fuels (e.g., H_2_), and for the inactivation of dangerous pathogens [10]. TiO_2_ in pure or modified form is the most popular and frequently tested photocatalyst. In nature, TiO_2_ exists in different crystal phases such as anatase, rutile, brookite, or TiO_2_(B). Among them, anatase and rutile have received particularly high attention [11,12,13,14]. Nanostructured TiO_2_ can be prepared by several chemical and physical routes [15,16]. For the chemical fabrication of TiO_2_ nanoparticles (NPs), the most frequently applied syntheses are the sol-gel and hydro- or solvothermal methods [17,18]. The sol-gel method is favored because it can be carried out without difficulties, and the composition of the metal oxide is well-controlled. The hydro- or solvothermal method requires pressure-tight vessels, however, the metal oxides obtained have high crystallinity even if the synthesis is performed at a relatively low temperature (typically <250 °C).

In addition to large-scale industrial utilization of TiO_2_ NPs, their agricultural applications are also being studied [19,20,21]. Nanomaterials-related investigations are extremely important and timely because of the rapid increase in the global population and the escalating environmental challenges due to climate change. Based on these aspects, novel and more effective agrochemicals are required in order to increase the yield in crop production. For example, nano-fertilizers and nano-pesticides with controlled releasing can be good alternatives instead of their conventional analogues [22,23]. Furthermore, because of the aforementioned reasons, nanomaterials that promote seed germination, stimulate plant growth, or induce greater stress tolerance in plants will be very useful in the near future for all humanity. Nevertheless, the available information on the potential phytotoxicity of nanomaterials including TiO_2_ is limited and frequently controversial in the literature. Both adverse [24] and beneficial [25,26] or non-toxic [27] impacts of TiO_2_ NPs on plants have been reported. The advantageous effects or potential phytotoxicity of nanomaterials are still debated [28]. This is likely because the diverse physicochemical properties of nanoparticles, hence their phytotoxicity, are related to a number of parameters, such as crystal phase, size, morphology, etc. Finally, nanotoxicity also depends on the plant species used in the model experiments [29]. The applied exposure method is a determinant factor for the uptake of nanoparticles. Application of nanomaterials in a hydroponic system, foliar exposure, or uptake from the soil will most certainly influence the results on phytotoxicity [30,31,32].

TiO_2_ NPs generate reactive oxygen species (ROS) in the presence of water and oxygen if they are excited by light with appropriate energy [9]. However, the photocatalytic activity of TiO_2_ NPs has been ignored in most toxicological studies even if they were used in foliar exposure under field conditions where the excitation of nanoparticles was ensured. Because the photocatalytic behavior of anatase and rutile is different [13,33,34], the type of the TiO_2_ polymorph (crystallinity) is of crucial importance from a phyto-toxicological point of view. In our previous study, we found that P25 TiO_2_, which is a mixture of anatase and rutile, was able to boost the biosynthesis of polyphenols and elevated the level of some mineral nutrients in grapevine leaves in a genotype-dependent manner [35]. We have also shown that plant pathogenic bacteria can be inactivated in vitro, using anatase NPs illuminated by UV-A light [36]. These previous results indicate that TiO_2_ may be utilized in plant protection, stimulating us to carry out further experiments using phase-pure nanocrystallites (NCs), in order to clarify the role of crystal structure in phytotoxicity.

In this work, we combined sol-gel synthesis and hydrothermal treatment, applying different synthesis conditions, to produce TiO_2_ NCs with very similar morphologies but different crystal phases, namely anatase, and rutile. After optical and structural characterization of phase-pure polymorphs, their photocatalytic activity was studied for the generation of OH^●^ and O_2_^●−^ radicals. The effects of phase-pure TiO_2_ NCs on photosynthesis and the elemental composition of grapevine leaves were investigated. The dose- and crystal phase-dependent phytotoxicity of TiO_2_ NCs were discussed.

## 2. Materials and Methods

### 2.1. Materials

2-Propanol (HiPerSolv CHROMANORM for HPLC, VWR, Radnor, PA, USA), acetonitrile (Promochem Optigrade, LGC Standards GmbH, Wesel, Germany) were gradient grade for liquid chromatography. TiCl_4_ (≥98%, Fluka Chemie AG, Buchs, Switzerland), NaOH (99.9%, Molar Chemicals Ltd., Halásztelek, Hungary), Dimethyl sulfoxide, DMSO (≥99.9%, Sigma-Aldrich, St. Louis, MO, USA) were used as received. 5,5-dimethyl-1-pyrroline *N*-oxide, DMPO, was synthesized as previously described [36], and it was freshly distilled before use. High purity deionized water was obtained by a LaboStar 7 TWF-UV (SG Wasseraufbereitung und Regenerierstation GmbH, Barsbüttel, Germany) system.

### 2.2. Synthesis

#### 2.2.1. Synthesis of Anatase Nanocrystals

A total of 50 mL of 2-propanol with small portions was carefully added to 22.90 g of TiCl_4_. After the violent exothermic reaction, 100 mL of deionized water was added to the mixture resulting in a transparent clear solution. Hydrolysis of the precursor was carried out by adding 250 mL of 1.5 M NaOH solution with a feed rate of ~5 mL min^−1^ during intensive magnetic stirring. The final pH was set to 6. The white dispersion obtained was stirred for a further 1 h, then centrifuged and washed with high-purity deionized water. The resulting sediment was re-dispersed in 1200 mL of deionized water and then treated hydrothermally at 180 °C for 1 h using a Multiwave 3000 (Anton Paar GmbH, Graz, Austria) microwave digestion system. The hydrothermal reaction was carried out according to the program displayed in Table 1. After hydrothermal treatment, the dispersions were centrifuged, and finally, the sediment was dried in air at 50 °C for 24 h. The sample was denoted MW-A.

#### 2.2.2. Synthesis of Rutile Nanocrystals

A total of 40 mL of 2-propanol with small portions was carefully added to 21.75 g of TiCl_4_. When the violent reaction was completed 150 mL of deionized water was added to the mixture. The resulting clear solution was left to stand at room temperature for three weeks without agitation. White solid precipitation was formed by the slow hydrolysis and condensation during aging. The precipitate was re-dispersed in the original medium by magnetic stirring, and the pH was set to 6 using NaOH solution. After further stirring for 1 h, the dispersion was centrifuged, then washed with deionized water, and finally re-dispersed in 650 mL of deionized water. Hereafter the synthesis was continued with the hydrothermal treatment with the same steps and heating program as we described for anatase nanocrystals. Finally, the sediment was dried at 50 °C in air for 24 h. The sample was denoted MW-R.

### 2.3. Experimental Site and Plant Material

Fifteen-year-old vines of *Vitis vinifera* L. cultivar ‘Cabernet Sauvignon’ were investigated under non-irrigated open-field conditions on the south-facing slopes of Mecsek Hills, in Hungary (latitude: 46°07′ N, longitude: 18°17′ E, 200 m a.s.l.). Vines were grafted on commonly used rootstock varieties ‘T5C’ (*Vitis berlandieri* × *Vitis riparia*). The soil was a Ramann-type brown forest soil mixed with clay formed on red sandstone covered by Pannonian sediment. Vines were grown with 3.5 × 1.2 m vine spacing with East-West row direction in vertical shoot positioned umbrella training system. Meteorological data were monitored using the WS600 automatic weather station (OTT HydroMet Fellbach GmbH, Fellbach, Germany). In 2020, the average annual temperature was 19.4 °C, the site receives 471 mm annual precipitation and 2067 h of sunshine.

### 2.4. Leaf Treatment

Grapevine leaves at the southern side of the canopy were treated with aqueous dispersions of MW-A and MW-R samples at the concentrations of 0.001% (10 mg/L), 0.01% (100 mg/L), 0.1% (1000 mg/L), and 1% (10,000 mg) on 26 August 2020. MW-A and MW-R dispersions were prepared using high-purity water without any additives. Prior to use, dispersions were homogenized by ultrasonication for 30 min. For treatments, sixteen individual trunks were selected using different shoots for each TiO_2_ concentration. Leaves of the individual trunk were treated with either MW-A or MW-R, without using both samples in one trunk. At the fourth or fifth node of shoots, leaves were masked along the main vein, splitting them into two parts, and then MW-A or MW-R dispersions were sprayed onto their uncovered adaxial leaf surface by using a simple hand sprayer. The treatments were carried out in five repetitions. Leaf samples were collected two weeks after the treatment. Removed leaves were put immediately into the water and then transported to the lab for analysis.

### 2.5. Extraction of Leaves for High-Performance Liquid Chromatography

The leaves were cut along the main vein for two parts and subsequently freeze-dried for 24 h using a ScanVac Cool Safe 110-4 Freeze Dryer (LaboGene ApS, Allerod, Denmark). The treated and untreated parts were grounded separately in a porcelain mortar, and then 50 mg of each was sonicated in 1 mL of 0.5% acetic acid in an ultrasonic bath for 30 min. The resulting suspension was centrifuged at 20,660× *g* for 10 min, and then the supernatant was filtered through a 0.22 μm Nylon syringe filter (FilterBio^®^, Labex Ltd., Budapest, Hungary).

### 2.6. Methods

Powder X-Ray Diffraction (XRD) patterns were acquired through the use of Cu Kα radiation with a Rigaku SmartLab X-ray diffractometer (Rigaku, Tokyo, Japan) operating at 40 kV and 150 mA. Bright-field transmission electron microscopy (BF-TEM) images have been acquired on a JEOL JEM-1011 instrument (JEOL Ltd., Tokyo, Japan) equipped with a thermionic W electron source and operated at an acceleration voltage of 100 kV. UV-vis Diffuse Reflectance measurements were performed on a thermo-Electron Evolution 500 double-beam spectrometer (Thermo Electron, Cambridge, UK) with an RSA-UC-40 diffuse reflectance accessory.

EPR spectra were recorded on a Miniscope MS200 spectrometer (Magnettech GMBH, Berlin, Germany) working with a modulation amplitude of 0.2 mT and a microwave power of 10.0 mW. Measurements were carried out in water (for OH^●^) or DMSO (for O_2_^●−^) at room temperature, containing 0.1 mg/mL of TiO_2_ NCs and 100 mM of freshly prepared DMPO. EPR spectra were recorded on the liquid obtained after 90, 120, 180, 300, and 600 s of photoirradiation of TiO_2_ dispersions containing DMPO. For quantification of DMPO-adducts, 10 μM 3-(hydroxymethyl)-2,2,5,5-tetramethyl-2,5-dihydro-1*H*-pyrrol-1-oxyl was used as external standard. All the measurements were repeated three times.

Micro- and macronutrients of the leaves were determined by means of inductively coupled plasma atomic emission spectroscopy (ICP-AES) using a Shimadzu ICPE-9000 instrument (Shimadzu Co., Tokyo, Japan). Prior to analysis, the lyophilized leaves were digested in 69 wt% HNO_3_ using a Multiwave 3000 (Anton Paar, Graz, Austria) microwave system.

PS II chlorophyll fluorescence measurements. Leaf photochemistry was characterized by chlorophyll fluorescence parameters using the MAXI version of Walz Imaging PAM (Heinz Walz GmbH, Effeltrich, Germany). After a 30-min-long dark adaptation period, detached grapevine leaves were placed under the detector while their petioles were wrapped in a wet tissue to avoid water loss. Minimal and maximal fluorescence yields (F_0_ and F_m_) were measured prior to and right after a saturating blue light pulse. The maximal quantum yield of photosystem II was calculated as F_v_/F_m_ = (F_m_ − F_0_)/F_m_ [37]. Application of TiO_2_ NCs at higher concentrations (0.1 or 1%) resulted in necrotic lesions with higher abundance, therefore all color-coded images recorded by the Imaging PAM were divided into 1 cm^2^ equal parts. Individual parts were considered as different data, and means were used for calculations.

HPLC analysis was performed on a Shimadzu Prominence UFLC system (Shimadzu Co., Kyoto, Japan) consisting of an on-line degassing unit (DGU-20A5R), pump (LC-20AD), column oven (CTO-20AC), autosampler (SIL-20AC HC), and fluorescence detector (RF-20A). Phenomenex Synergy^®^ 4 μm Hydro-RP 80 Å, 250 × 4.6 mm column was used; the column temperature was maintained at 20 °C. Chromatographic separation was achieved by isocratic elution using 0.5% (*v*/*v*) aqueous acetic acid as mobile phase. The flow rate of the mobile phase was kept at 1.5 mL min^−1^; the volume of injected sample was 10 μL. For fluorometric detection of pyridoxine, the excitation and emission wavelengths were at 290 and 395 nm, respectively. A calibration curve for quantification was obtained by measuring pyridoxine with known concentrations. The results were expressed in ng pyridoxine per mg dry weight (DW) of grapevine leaf.

### 2.7. Statistics

Statistical analysis was carried out in Excel^®^ (Microsoft Corp., Redmond, WA, USA). For the comparison of data obtained from the control and treated part of the same leaf, a two-sample paired Student’s t-test was applied. Data were expressed as a means ± standard deviation. Results were considered statistically significant at *p* < 0.05.

## 3. Results

### 3.1. Structural, Optical and Morphological Properties of Anatase and Rutile TiO_2_ NCs 

**Figure 1:**
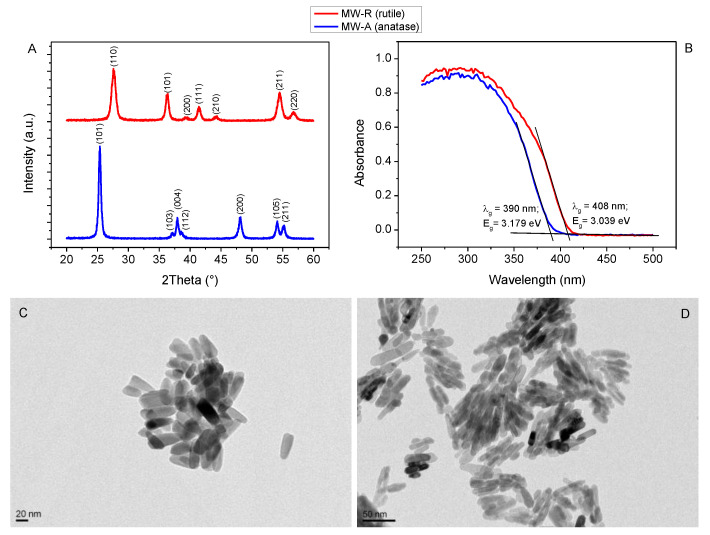
Comparison of structural, optical, and morphological properties: XRD patterns (**A**); and UV-Vis diffuse reflectance spectra (**B**) of MW-A (anatase) and MW-R (rutile TiO_2_) samples. Panel (**C**) and (**D**) show the TEM images of MW-A and MW-R, respectively.

X-ray diffraction (XRD) patterns of MW-A and MW-R powder samples are compared in Figure 1A, representing a sharp difference in their crystal structure. For the MW-A sample, characteristic diffraction peaks of anatase (JCPDS card No. 78-2486) were recorded which were assigned to (101), (103), (004), (112), (200), (105), and (211) lattice planes (Figure 1A bottom curve). For the MW-R sample (Figure 1A upper curve), rutile crystal phase with (110), (101), (200), (111), (210), (211), and (220) lattice planes were identified (JCPDS no. 21-1276). The XRD patterns revealed that both TiO_2_ nanocrystallites are phase-pure, consisting solely of either anatase or rutile. By using the Scherrer equation [38], the average crystallite sizes were calculated from the FWHM of (101) and (110) diffraction peaks of anatase and rutile, respectively. As a result, the average crystallite sizes of anatase and rutile nanocrystallites were found to be 22.6 and 13.4 nm, respectively.

Based on the diffuse reflectance UV-vis spectra (Figure 1B), the MW-A sample had a higher band gap energy (3.179 eV) than that of MW-R (3.039 eV). These values are in good agreement with previously published band gap energy of anatase and rutile TiO_2_ NCs [39]. Spectra show that both samples can be excited by either artificial UV-A radiation or the UV components of natural solar radiation.

By comparing the TEM images in Figure 1C,D, it can be established that the shape of the NCs in MW-A and MW-R samples was very similar. Moreover, both MW-A and MW-R samples showed uniform morphology and contained rod-like elongated nanocrystallites independently from their crystal phase. The length of anatase crystallites in MW-A was in the range ~25–60 nm (mean: 42 ± 6 nm), while their thickness was between ~10 and ~25 nm (mean: 15.2 ± 3 nm). The length of rutile crystallites in the MW-R sample varied between ~15 nm and ~60 nm (mean: 40 ± 10 nm) with a thickness of ~7–13 nm (mean: 10 ± 3 nm). The corresponding size distribution curves of NCs are presented in the Figure S1 of the Supplementary Materials. The aspect ratios were 2.8 and 4.0 for MW-A and MW-R NCs, respectively.

### 3.2. Crystal Phase-Dependent Formation of Reactive Oxygen Species

**Figure 2:**
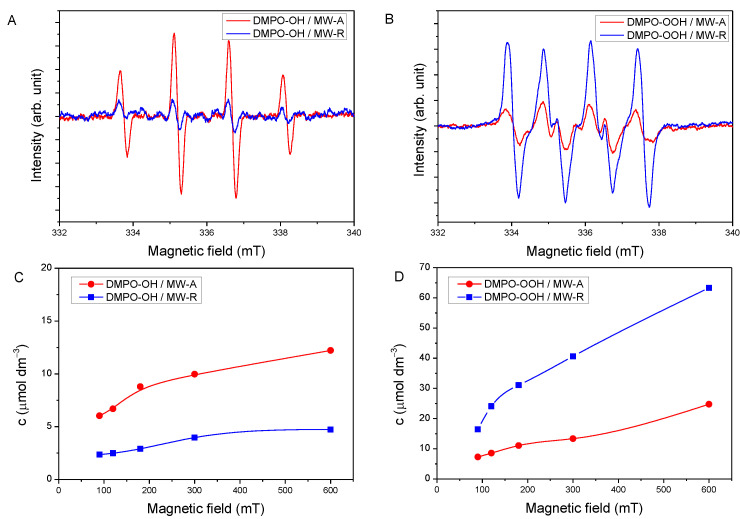
EPR spectra of DMPO-OH and DMPO-OOH adducts recorded in UV-irradiated dispersion of MW-A (**A**); and MW-R (**B**) at 300 s. Kinetic curves of the formation of DMPO-OH (**C**); and DMPO-OOH (**D**) adducts using MW-A and MW-R samples.

To characterize the nature of the ROS formed upon the irradiation of TiO_2_ NCs 5,5-dimethyl-pyrroline *N*-oxide (DMPO) was used as spin trap. DMPO can react with the OH^●^ and O_2_^●−^ radicals, forming paramagnetic spin-adducts, with a half-life of more than a minute, giving characteristic splitting patterns. In the aqueous dispersions of MW-A or MW-R, the characteristic 1:2:2:1 quartet EPR splitting pattern of DMPO-OH (Figure 2A), with hyperfine constants of a_N_ = 1.49 mT, a_H_ = 1.49 mT, were observed, meanwhile by using MW-A or MW-R in DMSO, DMPO-OOH spin adduct was detected with hyperfine constants of a_N_ = 1.37 mT, a_H_ = 1.0 mT (Figure 2B).

The concentration of DMPO-OH was 2.5–3 times higher in MW-A dispersion compared to MW-R, regardless of the time of photoirradiation (Figure 2C). The level of DMPO-OH adduct at 90 s was higher in MW-A dispersion compared to MW-R dispersion after 600 s UV-A irradiation (6.04 μM vs. 4.74 μM), proving that the surface of anatase is more favorable than that of rutile in the formation of hydroxyl radical (Figure 2C).

The kinetic curves of the formation of O_2_^●−^ radicals during UV-A irradiation are displayed in Figure 2D. By the comparison of MW-A and MW-R in DMSO, the DMPO-OOH level was 2.2 fold higher using MW-R (16.4 μM vs. 7.2 μM). Using the two photocatalysts, the difference in the O_2_^●−^ production activity remained 2–3-fold until 600 s irradiation.

### 3.3. Changes of the Level of Mineral Nutrients in Grapevine Leaves Induced by Anatase and Rutile TiO_2_ NCs

**Figure 3:**
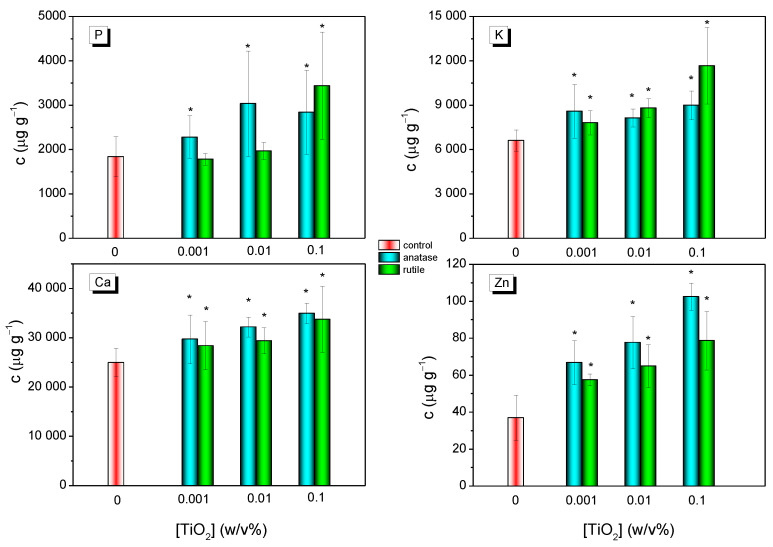
Level of P, K, Ca, and Zn elements in Cabernet Sauvignon leaf samples with and without MW-A or MW-R treatments using 0.001%, 0.01%, or 0.1% aqueous dispersions. * significant difference compared to control (*p* ≤ 0.05).

The concentrations of main macro- and microelements in the grapevine leaves were monitored by ICP-AES analyses. The level of macronutrients such as P, K, Ca, and Mg, moreover, microelements such as Fe, B, Mn, and Zn were studied. The main results are summarized in Figure 3. TiO_2_ NCs treatment significantly increased the level of P, K, Ca, and Zn even if the lowest TiO_2_ concentration (0.001 *w*/*v*%) was applied. One exception was observed for MW-R-treated samples, in which phosphorus level increased only in case of 0.1 *w*/*v*% dispersion. The average concentration of P using 0.1% MW-A and MW-R dispersions was 2840 and 3438 μg g^−1^, respectively. In contrast, 1841 μg g^−1^ of P was measured in control leaf parts. The level of K in the untreated samples was 6615 μg g^−1^, whereas it was 8996 and 11,660 μg g^−1^ in the 0.1 *w*/*v*% MW-A and MW-R-treated ones, respectively. The Ca level was found to be 24,976 μg g^−1^ in the untreated samples, while it was 34,951 and 33,718 μg g^−1^ in the 0.1 *w*/*v*% MW-A- and MW-R NCs-treated ones. Without TiO_2_ NCs treatment, the concentration of Mg was 1337 μg g^−1^ (not presented). Using 0.1 *w*/*v*% MW-A or MW-R dispersions, the concentration of Mg was 2926 or 2585 μg g^−1^ in the treated leaf parts. Treatments induced the Zn accumulation which increased gradually with increasing the TiO_2_ concentration. Control leaf parts contained Zn with an average concentration of 36.9 μg g^−1^, whereas it was 102.7 and 78.7 μg g^−1^ in the MW-A- and MW-R-treated ones, respectively. Mn concentration (not presented) was found to be higher in all MW-A-treated samples compared to control parts. However, MW-R did not induce Mn accumulation. Fe and B contents were also independent of the treatment.

### 3.4. Dose-Dependent Phytotoxicity of Anatase and Rutile TiO_2_ NCs

**Figure 4:**
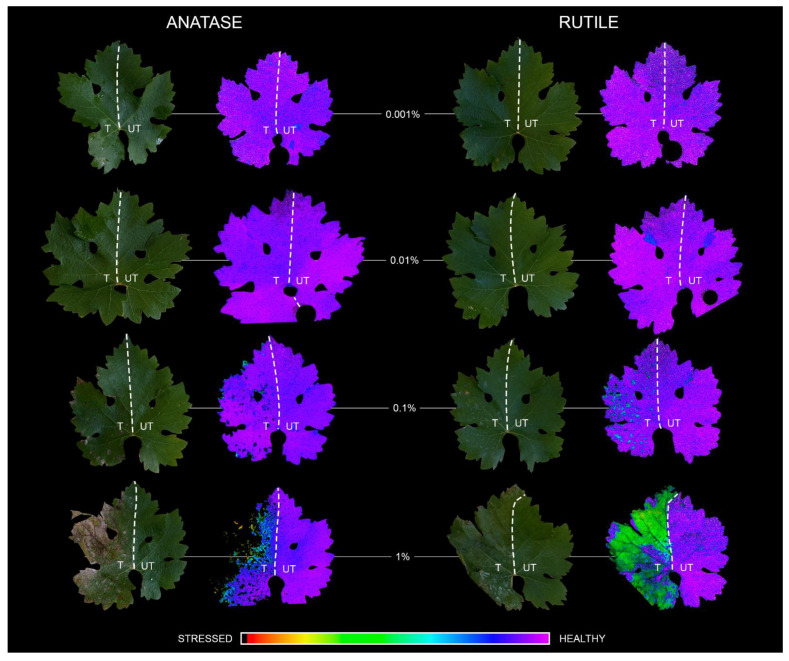
Digital and color-coded chlorophyll fluorescence images of anatase (**left pair**) and rutile (**right pair**) TiO_2_ NCs-treated Cabernet Sauvignon grapevine leaves along a concentration gradient (0.001, 0.01, 0.1, and 1 *w*/*v*%). TiO_2_ NCs treatments were carried out by masking the leaves along the midvein, which divided them into two parts (dashed line): treated side on the left (T), untreated control on the right (UT). The false-color coding refers to the maximal quantum yields of photosystem II (Fv/Fm). The color scale depicted at the bottom of the figure represents the range between stressed and healthy stages.

Photosystem (PS) II chlorophyll fluorescence parameters were used for assessing photosynthetic responses of TiO_2_ NCs-treated grapevine leaves (Figure 4.). The two applied TiO_2_ dispersions affected the photosynthesis quasi-similarly: Both anatase and rutile treatments caused a slight (2 and 1%) but significant increase of the maximal quantum yield of PS II (Fv/Fm) if applied in 0.001 *w*/*v*% concentration. That positive effect of rutile has remained in 0.01 *w*/*v*% concentration as well, while the same amount of anatase kept the treated leaf part unaffected. At elevated concentrations (0.1 or 1 *w*/*v*%), the distribution of TiO_2_ NCs on the leaf surface was not homogenous due to the manual spraying which resulted in localized stressed (rutile) or even necrotic (anatase) sites (Figure 4). Despite these necrotic sites, the rest of the leaf-part had only 2% (anatase) and 1% (rutile) average loss of leaf photochemistry in case of 0.1 *w*/*v*% TiO_2_ NCs treatment. That is suggesting that the Cabernet leaves were able to compensate for the adverse effect and were capable to acclimate to this concentration of dispersions. Notwithstanding, the localization of 1 *w*/*v*% TiO_2_ NCs is obvious, the Fv/Fm means significantly reduced by 16% (anatase) and 19% (rutile).

### 3.5. Anatase and Rutile TiO_2_ NCs Triggered the Antioxidant Defense System Enhancing the Level of Vitamin B_6_ in Grapevine Leaves

**Figure 5:**
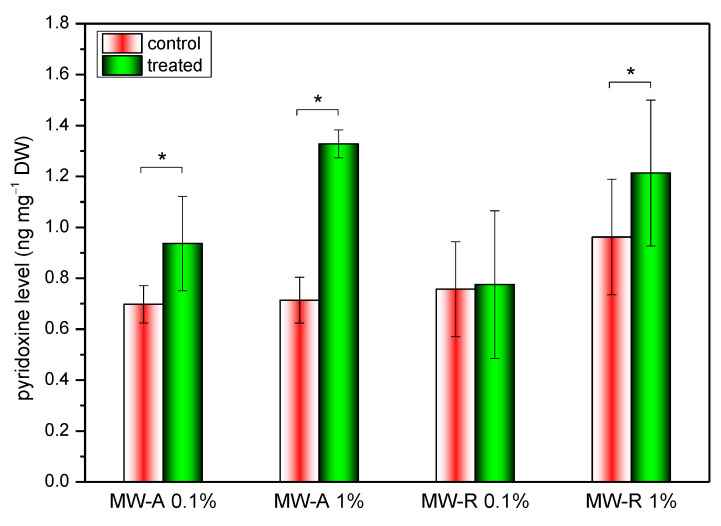
Comparison of the pyridoxine level in the control and TiO_2_ NCs-treated leaf parts. * significant difference compared to control (*p* ≤ 0.05).

The level of pyridoxine, the main vitamin B_6_ in grape leaves, was determined by HPLC-FLD analysis. Similar to the imaging-PAM investigations, leaves were divided into two different parts along the midvein, then the non-treated (control) and TiO_2_-treated leaf parts were used separately for the preparation of extracts and the analysis. The results obtained are shown in Figure 5. A significant increase of pyridoxine level was observed for the leaves treated with 0.1 *w*/*v*% and 1 *w*/*v*% MW-A dispersions. For MW-R TiO_2_ NCs samples, only 1% dispersion triggered the enhancement of pyridoxine level. The treatment with 1% MW-A increased pyridoxine content from 0.71 to 1.33 ng mg^−1^, while the application of 1% MW-R increased its level from 0.96 to 1.23 ng mg^−1^.

## 4. Discussion

In this work, we successfully synthesized TiO_2_ NCs with similar morphology but different crystal phases from the same precursor (TiCl_4_) by the combination of a sol-gel method and a hydrothermal post-treatment. The results show that our method is not only suitable for the production of phase-pure anatase and rutile NCs (Figure 1) but the morphology of the nanocrystallites can be controlled without the use of any additives such as surfactants or other structure-directing agents. One of the key factors of the phase-selective synthesis was the pH applied during the hydrolysis of the precursor. While higher pH favors the formation of anatase, rutile formation was preferred under strongly acidic conditions [40,41]. Besides pH, the rate of hydrolysis was also an important factor for controlling the crystal phase of TiO_2_ NCs. This is supported by the fact that the final pH was set to the same value (pH 6), but the crystal phase of the MW-A and MW-R samples was different. Based on these considerations, to produce anatase (MW-A sample) pH was raised relatively quickly (within 1 h) to 6 by adding NaOH solution. In contrast, for the preparation of rutile (MW-R sample), no NaOH was added to the precursor solution for one week, thereby the reaction medium remained extremely acidic (pH < 1) during hydrolysis and condensation. Furthermore, since the hydrolysis was faster in the case of MW-A, the reaction product, TiO_2_ × H_2_O hydrous gel, possessed very low crystallinity, requiring the use of a post-crystallization step. Hydrothermal treatment is an excellent method to increase the crystallinity of various metal oxides [42], and therefore, both as-prepared MW-A and MW-R samples were treated hydrothermally applying relatively low temperature and short reaction time (180 °C, 3 h). Tailoring the crystal phase is of great importance since the reactivity of the (photo)catalysts fundamentally depends on the crystal structure [43,44,45,46]. It has been reported that anatase exhibits higher photocatalytic activity than rutile [13]. However, other important parameters of the given polymorph, in particular the synthesis method, are frequently ignored. For example, rutile TiO_2_ can be prepared from anatase through calcination [47,48,49,50] which leads to the aggregation of the primary particles and concomitant decrease of the specific surface area. Consequently, rutile TiO_2_ prepared by calcination usually shows low photocatalytic activity. In contrast, our samples synthesized with a wet-chemical route without the application of an annealing step did not show irreversible aggregation of primary particles as revealed by TEM investigation (Figure 1C,D). Besides the crystal phase, the shape of the nanocrystallites also plays a key role in (photo)catalytic activity. The importance of particle morphology and crystal facets has been highlighted by several studies [51,52,53]. Similar to our MW-A and MW-R samples, titania-based nanomaterials with elongated morphology are of great interest and have been widely investigated for photocatalytic applications [54].

The EPR investigation confirmed the formation of OH^●^ and O_2_^●−^ on anatase- and rutile TiO_2_ NCs (Figure 2). However, the ROS profile was clearly different for the two polymorphs. A higher level of OH^●^ formed using anatase (MW-A) than rutile (MW-R) under the same experimental conditions (Figure 2C). OH^●^ is one of the most reactive ROS, thus the superior photocatalytic activity of anatase is frequently explained by its greater OH^●^ producing activity [13,55]. Nevertheless, our MW-R sample proved to be a highly active O_2_^●−^ producer (Figure 2D) under UV-irradiation, which can be attributed to the better O_2_ adsorption ability of rutile. Since rutile TiO_2_ is a stronger reducer than anatase, O_2_^●−^ is easily formed on rutile as a result of O_2_ reduction [56,57]. ROS as signaling molecules play a crucial role in various physiological processes; they are essential for life [58,59]. ROS including OH^●^ and O_2_^●−^ are unavoidably formed as a normal product of aerobic metabolism [60]. In planta, their formations are inevitable in chloroplasts, mitochondria, peroxisomes or plasma membranes, and as a byproduct of cell metabolism, and in different cellular compartments [61]. ROS participate in numerous plant stress responses [62]. Based on our EPR investigations, the formation of both OH^●^ and O_2_^●−^ is strongly expected in anatase- or rutile-treated grapevine leaves exposed to natural sunlight containing ultraviolet components. Thus, the grapevine leaves are not only exposed to TiO_2_ NCs, but ROS formed in the photocatalytic reaction should also be considered. We have already demonstrated that the levels of micro- and macro-elements are increased in grapevine leaves in response to Degussa P25 treatment [35,63]. Degussa P25 consists of anatase (88 wt%) and rutile (12wt%), and here we show that both anatase and rutile treatments are able to upregulate mineral nutrients on their own (Figure 3). A comparison of the two polymorphs in this study showed that anatase promoted the accumulation of P and Zn to a higher extent than rutile. Meanwhile, the increase of Ca and K levels were not significantly dependent on the polymorph nature. Changes in the elemental composition have been already observed at the lowest dosage of TiO_2_ (0.001%). Furthermore, the accumulation of nutrients slightly increased when we applied MW-A or MW-R at higher concentrations, but the exact mechanisms responsible for the selective accumulation of nutrients are yet to be explored. In fact, we are not aware of any other study in which the relation between the foliar application of TiO_2_ NPs and the mineral nutrient content of leaves was investigated. In turn, TiO_2_ NPs delivered to the soil and then taken up through the root also significantly increased P (by 34%) and K (by 35%) contents in cucumber fruits [64]. For the increased nutrient content, another plausible explanation can be that some Ti compounds at low concentrations stimulate certain enzymes and thus promote nutrient uptake. Although this effect has not yet been described for TiO_2_ NPs, the beneficial impact of water-soluble Ti chelates on plants (such as strengthening stress tolerance, improving crop yield, and enhancing chlorophyll content and photosynthesis) are well documented [65]. Finally, a further plausible explanation for the elevated elements could be the photo-catalytically induced ROS, which regulates the transport and mobilization of nutrients through the activation of signaling pathways [66,67]. H_2_O_2_ and OH^●^ are the major activating species for the ion channels [68,69]. ROS are involved in plant stress responses and play crucial roles in the generation of cytoplasmic Ca^2+^ signals [66]. Also, ROS can directly activate the K^+^ channels AtGORK and AtSKOR, which are responsible for K^+^ efflux from cells during stress [66].

Under environmentally balanced conditions, the level of ROS is well-controlled by the enzymatic and non-enzymatic defense systems. Under oxidative stress, ROS are overproduced, and thereby the delicate equilibrium between oxidants and antioxidants is harmed. The predominance of the oxidative species can be detrimental or fatal to the cell causing oxidative damage of DNA, lipids, etc., or ultimately cell death. Consistent with these, our chlorophyll fluorescence measurements showed that the phytotoxicity of TiO_2_ is dose-dependent (Figure 4). TiO_2_ NPs are able to promote the conversion of inorganic forms of nitrogen such as ammonium or nitrate to organic nitrogen through the upregulation of nitrate reductase enzyme and hence support chlorophyll metabolism in leaves [70,71,72]. Elevated chlorophyll content orchestrated by the TiO_2_ NCs can result in more effective photosynthetic machinery [73]. In this study, we experienced that this photosynthesis-supporting nature of TiO_2_ NCs was dependent on the applied concentrations. On leaves treated by lower concentrations (0.001% or 0.01%) of either rutile or anatase, we also recorded elevated maximal quantum yields of PS II (Fv/Fm), but in higher concentrations (0.1% or 1%), both TiO_2_ NCs become phytotoxic due to the excessive production of photogenerated charge carriers (electrons and holes), OH^•^ and O_2_^●−^. This was supported by the presence of necrotic lesions on 0.1% and 1% TiO_2_-treated leaves, as an obvious consequence of the predominance of oxidative processes. Phytotoxicity of TiO_2_ was found to be polymorph-dependent. The difference in the ROS profile of the two TiO_2_ polymorphs revealed by EPR investigations is of great physiological importance. The greater phytotoxicity of anatase can be related to the fact that this polymorph produced extremely reactive OH^•^ with a higher concentration than rutile. On the other hand, plants have no enzymatic defense system against OH^•^ radicals, whereas O_2_^●−^ can be scavenged via both enzymatic and non-enzymatic ways [74]. To mitigate the adverse effects of oxidative stress, plants also employ non-enzymatic defense producing various antioxidants such as polyphenols or vitamins [75]. Vitamin B_6_ (pyridoxine) is an important participant in plant stress responses. In addition to scavenging ROS directly, it acts as an essential cofactor of several enzyme reactions, for example, in the biosynthesis of many antioxidant enzymes [76] or non-enzymes [77]. Colinas and Fitzpatrick [78] reported strong interaction between nitrogen supply and vitamin B_6_ formation in thale cress, lack of N led to malfunctioning pyridoxine synthesis. Based on that, our results suggest that TiO_2_-accelerated N-uptake caused elevated pyridoxine concentrations in grapevine leaves. Anatase treatments enhanced the pyridoxine biosynthesis even in a lower concentration. For rutile, only 1% dispersion provoked significantly higher pyridoxine levels. Overall, according to Imaging-PAM and HPLC measurements, anatase treatments resulted in more severe stress for Cabernet Sauvignon leaves than rutile TiO_2_ NCs, probably because of the more intense formation of OH^•^ caused by the anatase. As far as we know, this is the first study in which the profile of TiO_2_ NCs-induced ROS is considered from a phytotoxic point of view.

## 5. Conclusions

The impact of TiO_2_ NCs on grapevine leaves can be either beneficial or adversarial. Our results clearly show that both anatase and rutile NPs affect leaf photochemistry and mineral nutrient in a dose-dependent manner. While TiO_2_ NCs at low concentrations (0.001%, 10 mg/L) improved the photosynthetic performance of grapevine leaves, application at higher concentrations (0.1%, 1000 mg/L) were detrimental, causing severe photocatalytic stress, which led to serious tissue damages in the leaves. Treatment with rutile TiO_2_ NCs dispersion at 1% (10,000 mg/L) concentration made PS II activity strongly inhibited, while the anatase form caused completely damaged sites on a leaf. Both polymorphs increased the mineral nutrient content, even at low TiO_2_ concentrations (10 mg/L) without damaging leaf tissues. The two polymorphs’ different ability to generate photooxidative damage of leaf tissues can be explained by the different ROS profiles: anatase produces OH^●^ in higher intensity, rutile is rather a source of O_2_^●−^. The elevated pyridoxine level indicated that hydroxyl radical productive anatase NPs cause higher stress.

To sum up, our synthesis methods are suitable for the production of phase-pure anatase and rutile TiO_2_ NCs with rod-like morphology. The presented syntheses protocols will be useful tools for further studies investigating the (phyto)toxicity of anatase and rutile TiO_2_. Our results suggest that TiO_2_ NCs as potential elicitors in low concentrations (10–100 mg/L) can be utilized in agricultural applications to enhance the photosynthetic performance, mineral nutrient level, and to aid in plant defense against abiotic and biotic challenges. Further studies are necessary to clarify these issues in detail.

## Figures and Tables

**Table 1:** Heating program of microwave digestion system for hydrothermal treatment of TiO_2_ dispersions.

Steps	Power (W)	Ramp (min)	Hold (min)	Fan
1	300	5	5	1
2	1000	10	5	1
3	1400	10	60	1
4	0	-	15	3

## Data Availability

Those unpublished data which may provide additional information in order to understand the present research are accessible from the corresponding author.

## Supplementary Materials

The following are available online at http://www.mdpi.com/2079-4991/12/3/483/s1, Figure S1: The size distribution of anatase and rutile nanocrystals.
