# Supplementary material for: Phase-Selective Synthesis of Anatase and Rutile TiO2 Nanocrystals and Their Impacts on Grapevine Leaves: Accumulation of Mineral Nutrients and Triggering the Plant Defense
Source: Nanomaterials (Basel). 2022 Jan 29;12(3):483. doi: 10.3390/nano12030483 (PMC8838626; doi:10.3390/nano12030483)
Supplement: Supplementary file 1 [file nanomaterials-12-00483-s001.zip › nanomaterials-1566118-supplementary.pdf]

nanomaterials

Supplementary Information

Phase-Selective Synthesis of Anatase and Rutile TiO_2 Nanocrystals and Their Impacts on Grapevine Leaves: Accumulation of Mineral Nutrients and Triggering the Plant Defense

László Kőrösi [1,*], Balázs Bognár [2], Gyula Czégény [3] and Simone Lauciello [4]

1. Research Institute for Viticulture and Oenology, University of Pécs, Pázmány P. u. 4, Pécs H-7634, Hungary
2. Institute of Organic and Medicinal Chemistry, Faculty of Pharmacy, University of Pécs, Szigeti st. 12, H-7624 Pécs, Hungary; balazs.bognar@aok.pte.hu
3. Department of Plant Biology, University of Pécs, Ifjúság u. 6, H-7624 Pécs, Hungary; czegeny@gamma.ttk.pte.hu
4. Electron Microscopy Facility, Istituto Italiano di Tecnologia, Via Morego 30, 16163 Genova, Italy; Simone.Lauciello@iit.it

* Correspondence: korosi.laszlo@pte.hu

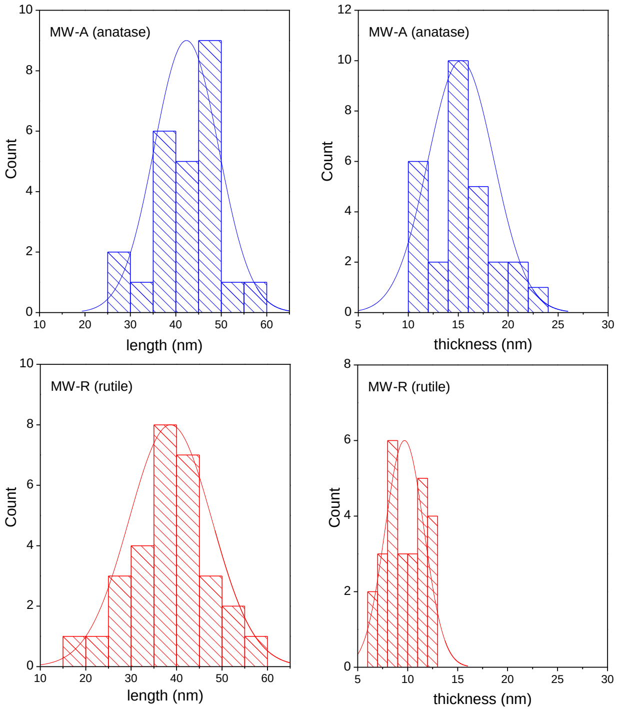

Figure S1. Size distribution of MW-A (anatase) and MW-R (rutile TiO_2) NCs.

in the direction of length and thickness.
